# Supplementary material for: Educational considerations for health professionals to effectively work with clients with complex regional pain syndrome
Source: PLoS One. 2022 Jun 13;17(6):e0269337. doi: 10.1371/journal.pone.0269337 (PMC9191715; doi:10.1371/journal.pone.0269337)
Supplement: Attachment — Submitted filename: Response to reviewers.docx [file pone.0269337.s003.docx]

Stefano Triberti, Ph.D.
Academic Editor

PloS One

8^th^ March 2022

Dear Professor Triberti,

Thank you for the opportunity to revise and resubmit the manuscript “Educational considerations for health practitioners and CRPS patient engagement” written by Dr Colleen Johnston-Devin, Professor Marion Gray, Associate Professor Florin Oprescu, and Professor Marianne Wallis.

We appreciate the effort involved in providing the review and submit now the COREQ checklist, Response to reviewers, Revised manuscript with track changes and the clean copy Manuscript. We do not wish to make changes to the financial disclosure.

The minimal data set for this work can be accessed via [https://doi.org/10.25907/00041](https://protect-au.mimecast.com/s/tkn1CwVLY6fOONQNi1Wmt4?domain=doi.org). Consent was not asked of participants for permission to make the interview transcripts publicly available, however the information provided is enough for the interviews to be repeated with different participants. Even though participants all chose a name to be used for the research, they are potentially identifiable if their transcripts were available due to detail provided in some interviews.

As suggested, the poem in the Supporting Information has been removed.

**Comments to the Author**

1. Is the manuscript technically sound, and do the data support the conclusions?

The manuscript must describe a technically sound piece of scientific research with data that supports the conclusions. Experiments must have been conducted rigorously, with appropriate controls, replication, and sample sizes. The conclusions must be drawn appropriately based on the data presented.

Reviewer #1: Yes

Reviewer #2: Partly

2. Has the statistical analysis been performed appropriately and rigorously?

Reviewer #1: N/A

Reviewer #2: N/A

3. Have the authors made all data underlying the findings in their manuscript fully available?

The [PLOS Data policy](https://protect-au.mimecast.com/s/LsSkCE8kNLHPDXyoT4rsYz?domain=plosone.org) requires authors to make all data underlying the findings described in their manuscript fully available without restriction, with rare exception (please refer to the Data Availability Statement in the manuscript PDF file). The data should be provided as part of the manuscript or its supporting information, or deposited to a public repository. For example, in addition to summary statistics, the data points behind means, medians and variance measures should be available. If there are restrictions on publicly sharing data—e.g. participant privacy or use of data from a third party—those must be specified.

Reviewer #1: Yes

Reviewer #2: Yes

4. Is the manuscript presented in an intelligible fashion and written in standard English?

PLOS ONE does not copyedit accepted manuscripts, so the language in submitted articles must be clear, correct, and unambiguous. Any typographical or grammatical errors should be corrected at revision, so please note any specific errors here.

Reviewer #1: Yes

Reviewer #2: Yes

| **Reviewer’s comment** | **Authors response** | **Page (clean version)** |
| --- | --- | --- |
| **Reviewer #1:** Please see the attached document comments and respond accordingly. Did you include unpublished studies? | Thank you for your detailed suggestions to improve this manuscript. No unpublished studies were included. |  |
| - Some descriptors were not used in the search and may have compromised and search strategy. | The initial literature search was rigorously undertaken. Updated articles have been included. | 3 |
| Exclusion criteria. The exclusion criteria are not clear. I didn't understand the first sentence I suggest revision of English | Participants did not have to prove their diagnosis. This is the same as previous research. | 6 |
| Please include an introduction in your abstract. | Introduction included as suggested | 2 |
| The introduction looks like a problem. You need to rewrite it. | Rewritten as suggested | 3 |
| The flow or the coherence needs arrangement | Agree with the reviewer. The paragraphs have been rearranged to improve clarity. | 3 |
| This should come first and should be introduced at the above | Thank you for this suggestion. Changed as suggested. | 3 |
| you don't have to explain about this in the design section. All you have to mention is what design you followed | Sentence moved to Rigour section. “Heuristics was included in the design to allow data from the lived experience of the first author, who has been diagnosed with CRPS, to be included as data without it dominating the research”. | 8 |
| How about the other language speakers? Do you think it is ethical to exclude the others as they have a lot to say about their condition. | We agree that using only English speakers may be a limitation however as the interviewer does not speak any other languages inclusion of non-English speakers was beyond the scope of the research. Sentence about future research including non-English speakers has been included. | 22 |
| Is it a data quality control or data collection procudure? you should make an arrangment to it | Data from pilot interviews were included in the analysis and therefore pilot interviews are included under the Data Collection heading. | 7 |
| Was it manual or assisted by a software ? Not clear for me. | Sentence amended for clarity. The data were analysed manually by following the steps outlined by van Manen. | 8 |
| How about the transferability issue and other rigour methods you haven't included and addressed them. | The data analysis and rigour sections have been expanded to address this comment. | 8 |
| You can put the themes in Thematic map rather than listing in a bullets | We attempted this but bullet points were clearer. | 9 |
| It is not necessary to add quotes and | Explanation provided in Rigour section and commencement of Findings. | 8, 9 |
| We have to know who said this. and you better put it in a | This quote was from the first author who did not choose a pseudonym for transparency. | 10 |
| Why do you mention the names | Pseudonyms were used so readers could gain a sense of hearing the voice of a person, and identifying with them, rather than a number which evokes less emotional response. | 8 |
| You are taking quotes as a theme but theme is a concept. you need to change it | In this instance, the quotes provided the inspiration for naming of the theme. | 12 |
| Very spliced topic... Please make it a theme | The findings section has been expanded to address the review comments and a new theme created as suggested. | 21 |
| You better make your discussion based on themes | As per Reviewer 2, this has been revised and made clearer. | 9 |
| ????? | Table 2 has been removed. |  |
| The recommendation should come after your conclusion | This has been moved as suggested. | 24 |
| The limitation and your way to manage it should be clear | Information has been added to this section to address this comment. | 22, 23 |
|  |  |  |
| **Reviewer #2**: Manuscript title: “Educational considerations for health professionals to effectively work with clients with complex regional pain syndrome”  Overall comment This manuscript presents a study which aimed to “To determine what people living with complex regional pain syndrome (CRPS) think health professionals should know about their condition to provide appropriate care”. It employed a heuristic, hermeneutic phenomenological study, and used semi-structured interviews with 17 participants living with CRPS. The manuscript is well written, interesting, and relevant. I applaud the authors for using a qualitative approach to investigate the experiences of living with a syndrome that is very challenging for patients and health professionals.  There are some issues to consider that would, in my opinion, improve the manuscript. Here are some specific comments: | Thank you. We appreciate the guidance to improve the manuscript. |  |
| Background The authors mention that “there is no global mandated content level of pain education for undergraduate health professionals` students…”. In my opinion, this sentence needs clarification since the International Association for the Study of Pian (IASP) has developed work in this area. I suggest a revision, as this may also ensure coherence between the information presented in the introduction and discussion sections. | Thank you for this suggestion. The information has been removed from the introduction. |  |
| Methods Sample/ Participants Information about participants (e.g. mean time lived with CRPS or mean time from symptom onset to formal diagnosis) is briefly presented and table 1 is indicated for more detailed data. I suggest the inclusion of a brief sentence about health professionals since it is also included in the table but not mention in the main text. | Added as suggested. | 6, 7 |
| Data analysis The link from reference (20) is not working. | The link has been fixed. | 8 |
| Findings Information presented in this section is quite interesting. Since the authors have used a hermeneutic phenomenological approach, I would expect a deeper interpretation of the data. On several occasions the manuscript is focused on merely describing what the participants had said and a quotation is presented. For example: “Alice found people touched her legs more often once she began using a wheelchair but stated, “I’ve seen them do it to other people in wheelchairs”. Sharon’s concern was the length of time it took her pain to settle after uninvited touch. If you walk past me the wrong way and you touch that arm, that’s a big deal for weeks, months, hours if you’re lucky. But people just can’t grasp the concept of how much pain. I reckon that’s the biggest hurdle. Sharon” Additionally, considering the hermeneutic phenomenological approach I would not expect an idiographic focus on this analysis. The main text presented in the findings section calls attention to the individual`s participations, which would be expected in an interpretative phenomenological analysis (that integrates phenomenology, hermeneutic and idiography).  In my opinion, these two aspects may compromise the quality of the manuscript. I strongly recommend a revision of this section. | Thank you, we agree with the reviewer. The findings section has been expanded to address the review comments. | 9 - 22 |
| Discussion The authors discussed the main findings of this study and introduced some references from relevant research. I recommend the revision of two aspects (the introduction of a poem and the anecdotal evidence on veterinary science) explored in the next paragraphs. I believe that the poem has provided a good inspiration and promoted reflection, which is very important for researchers working with qualitative data. However, in the discussion section the readers would expect a discussion focused on the relation between this study`s findings and previous research. I think the presentation of this poem does not add value for the discussion itself. The authors mentioned that “anecdotal evidence exists supporting the notion that veterinary science undergraduate programs contain more pain content than that of a medical degree”. Since this is based on anecdotal evidence, I don`t think this information adds value to this discussion. The following sentence (“Research suggest that limited pain content is taught in undergraduate health sciences courses (5,31))” is clear, based on previous research and may be used without the anecdotal evidence. | Thank you for this suggestion. The poem has been removed. The anecdotal veterinary science information has also been removed. |  |
| Table 2. The authors could cite the reference. Is this table really needed? | Table removed as suggested and the reference cited. | 21 |

Yours sincerely,

Colleen Johnston-Devin

**Dr Colleen Johnston-Devin, RN, BN(post reg), GCAdv Paed Nurs, GCTAE, PhD**
Assistant Head of Course, Master of Clinical Nursing | Lecturer

School of Nursing, Midwifery & Social Sciences

Higher Education Division
CQUniversity Australia **P** +61 07 5440 7081 **| X** 47081 **| E**  [c.johnston-devin@cqu.edu.au](mailto:c.johnston-devin@cqu.edu.au)
