# Supplementary material for: Educational considerations for health professionals to effectively work with clients with complex regional pain syndrome
Source: PLoS One. 2022 Jun 13;17(6):e0269337. doi: 10.1371/journal.pone.0269337 (PMC9191715; doi:10.1371/journal.pone.0269337)
Supplement: Attachment — Submitted filename: Response to reviewers.docx [file pone.0269337.s005.docx]

Stefano Triberti, Ph.D.
Academic Editor

PloS One

9^th^ May 2022

Dear Professor Triberti,

Thank you again for the opportunity to revise and resubmit the manuscript “Educational considerations for health practitioners and CRPS patient engagement” written by Dr Colleen Johnston-Devin, Professor Marion Gray, Associate Professor Florin Oprescu, and Professor Marianne Wallis.

We appreciate the effort involved in providing the review and submit the Response to reviewers, Revised manuscript with track changes and the clean copy Manuscript to you. We do not wish to make changes to the financial disclosure.

Thank you for your comment on the idiographic focus missing. You are correct. This study used phenomenology as described by van Manen (1990) who states, “phenomenology attempts to explicate the meanings as we live them in our everyday existence” (p.11). Our understanding is that nomothetic and idiographic perspectives are often used in psychology, but also used in ethnography and sociology. Nomothetic is usually quantitative and seeks to generalise. Although generalisation is not an aim of phenomenology (Crotty, 1996), it illuminates the essence of the phenomenon and the general structure of meaning which is a commonality through the experiences of each participant (van Wijngaarden et al., 2017). Idiographic approaches are usually qualitative and seek to explore what characteristics of a group separate it from another group. This was not the intent of this research and thus not discussed in this paper.

Crotty, M. (1996). *Phenomenology and nursing research*. South Melbourne, Victoria: Churchill Livingstone.

van Manen, M. (1990). *Researching lived experience: Human science for an action sensitive pedagogy*. New York: State University of New York Press.

van Wijngaarden, E., Meide, H., & Dahlberg, K. (2017). Researching health care as a meaningful practice: Toward a nondualistic view on evidence for qualitative research. *Qualitative Health Research, 27*(11), 1738-1747. doi:10.1177/104973231771113

**Comments to the Author**

1. If the authors have adequately addressed your comments raised in a previous round of review and you feel that this manuscript is now acceptable for publication, you may indicate that here to bypass the “Comments to the Author” section, enter your conflict of interest statement in the “Confidential to Editor” section, and submit your "Accept" recommendation.

Reviewer #1: All comments have been addressed

Reviewer #2: (No Response)

6. Review Comments to the Author

Please use the space provided to explain your answers to the questions above. You may also include additional comments for the author, including concerns about dual publication, research ethics, or publication ethics. (Please upload your review as an attachment if it exceeds 20,000 characters)

Reviewer #1: (No Response)

Reviewer #2:

| Reviewer Comment | Author Response |
| --- | --- |
| Dear Authors Thanks for the submission of a new version with ammendments. In my opinion, the changes on introduction and methods have improved the quality of the paper. | Thank you |
| However, the same has not happened in the findings and discussion sections. Although there has been an attempt to improve the depth of the interpretation, the problem related to the emphasis on the individuals` participation remains the same. As I mentioned before, I would expect an idiographic element in an interpretative phenomenological analysis. The authors have replied that they agree with this idea but have not made changes to address this aspect and have not present a justification to do so. In my opinion, this still compromises the quality of the paper. | The findings section was expanded in line with the previous review comments. However, this research did not utilise IPA. This study used phenomenology as described by van Manen (1990) who states, “phenomenology attempts to explicate the meanings as we live them in our everyday existence” (p.11). Our understanding is that nomothetic and idiographic perspectives are often used in psychology, but also used in ethnography and sociology. Nomothetic is usually quantitative and seeks to generalise. Although generalisation is not an aim of phenomenology (Crotty, 1996), it illuminates the essence of the phenomenon and the general structure of meaning which is a commonality through the experiences of each participant (van Wijngaarden et al., 2017). Idiographic approaches are usually qualitative and seek to explore what characteristics of a group separate it from another group. This was outside the scope of this research and this paper.  The Design section (Page 5) has been amended for clarity:  “The hermeneutic phenomenological design chosen was informed by van Manen [16]. Acknowledging that the “(phenomenological) facts of lived experience are already meaningfully (hermeneutically) experienced” [16 p181], such research aims to describe, understand and interpret the experiences of the participants [17].This approach to phenomenology was chosen due to it using descriptions of lived experiences as data to describe the essence of a phenomenon [16]. This article reports on the data related to what health professionals should know about CRPS. The lived experience of living with CRPS is presented elsewhere [18]”.    Changes were previously also made in the Rigour section (Page 10) with the inclusion of the following two sentences:  “Essential and incidental themes in the data arose through the isolation of thematic statements and consensus agreements of the authors and the use of representative quotations from participants also adds transparency and trustworthiness to illustrate the interpretation of the findings presented (34). In the writing of the findings, readers can then judge the credibility and possible generalisability, or transferability to their own settings [34]”. |
| Additionally, the new version presents the findings together with discussion in the same section. This approach does not help the reader to follow the study and limits the depth of information presented in the findings and discussion sections. I would recomend the presentation of findings and discussion in different sections, as well as, the revision of findings in line with my previous comment (idiographic element). | The Findings and Discussion sections were combined because we misinterpreted the reviewers’ comment. These sections have again been separated. |

We thank the reviewers for the comments and contributions to improving the manuscript.

Yours sincerely,

Colleen Johnston-Devin

Dr Colleen Johnston-Devin, RN, BN(post reg), GCAdv Paed Nurs, GCTAE, PhD

Assistant Head of Course, Master of Clinical Nursing | Lecturer

School of Nursing, Midwifery & Social Sciences

Higher Education Division, CQUniversity Australia

P +61 07 5440 7081 | X 47081 | E c.johnston-devin@cqu.edu.au
